# The Anticipated Positive Psychosocial Impact of Present Web-Based E-Health Services and Future Mobile Health Applications: An Investigation among Older Swedes

**DOI:** 10.1155/2013/509198

**Published:** 2013-12-03

**Authors:** S. Wiklund Axelsson, L. Nyberg, A. Näslund, A. Melander Wikman

**Affiliations:** Department of Health Sciences, Luleå University of Technology, 971 87 Luleå, Sweden

## Abstract

This study investigates the anticipated psychosocial impact of present web-based e-health services and future mobile health applications among older Swedes. Random sample's of Swedish citizens aged 55 years old and older were given a survey containing two different e-health scenarios which respondents rated according to their anticipated psychosocial impact by means of the PIADS instrument. Results consistently demonstrated the positive anticipation of psychosocial impacts for both scenarios. The future mobile health applications scored more positively than the present web-based e-health services. An increase in age correlated positively to lower impact scores. These findings indicate that from a psychosocial perspective, web-based e-health services and mobile health applications are likely to positively impact quality of life. This knowledge can be helpful when tailoring and implementing e-health services that are directed to older people.

## 1. Introduction

The world is facing an increasingly aging population which is presently placing heavy demand on health care services, and this continues into the future [1]. There are growing expectations that e-health will be the solution for these demands. E-health refers to “tools and services using information and communication technologies (ICTs) that can improve prevention, diagnosis, treatment, monitoring, and management and can benefit the entire community by improving access to care and quality of care and by making the health sector more efficient” [2]. The goal is to make e-health both more user-friendly and thus more widely accepted by involving patients in strategy, design, and implementation, as well as supporting the general increase in quality of life [2, 3].

European countries, such as Norway, Denmark, Germany, Greece, and Portugal, show steady development in using the Internet as a source for health information [4]. In Sweden, it is possible for citizens nationwide to use web-based e-health services offered by Swedish public health care providers to receive general e-health information online [5], receive personalized web-based e-health services (e.g., online e-prescription renewal), ask their doctors questions online, obtain medical devices, and reschedule doctor appointments [6, 7]. The next generation of e-health systems is mobile health applications that are “considered as the strongest contribution for the next generation e-health systems” [8]. These applications act closely with an individual and “focus on serving the needs of the user by providing widespread access to relevant information and/or remote data capture, thus eliminating the need for the user to be physically linked to a network or restricted to a specific geographic location” [9].

E-health has been recommended for supporting the health conditions of older adults [3]. For older adults, health is closely linked to aspects of quality of life, such as psychological well-being, independence, mobility, safety, and social involvement [10–13]. In turn, these aspects are impacted by personal and environmental factors that are equated with psychosocial factors [14] that are often challenged by health conditions which usually worsen with age [15]. Several self-reported evaluations of e-health services from the perspective of patients show a low effect on health related quality of life and psychological outcomes [16].

Clearly, investigating what impact e-health has on psychosocial issues among older adults matters to understanding how e-health can be supportive or counteractive to the health of older adults. In this sense, the full use and adoption of technology are related to its role, perceived usefulness, and meaning in an individualized context [17]. Benefits and lack of benefits affect the motivation for the introduction of new technology innovation among older adults [18]. In a previous study [19], we found a low degree of e-health service use among older adults in Sweden despite the extensive development of these services. The objective of this study was thus to investigate the anticipated psychosocial impact from present web-based-e-health services and future mobile health applications among older Swedes.

## 2. Methods

This study implemented a cross-sectional survey based on two scenarios, the Psychosocial Impact of Assistive Devices (PIADS) questionnaire and also background questions. The survey was distributed by post, and respondents answered the survey during telephone interviews or filled them in at home and returned by post. The study was approved by the Regional Ethical Review Board in Umeå, Sweden [Ref. no. 2594-10].

### 2.1. Sample

**Figure 1:**
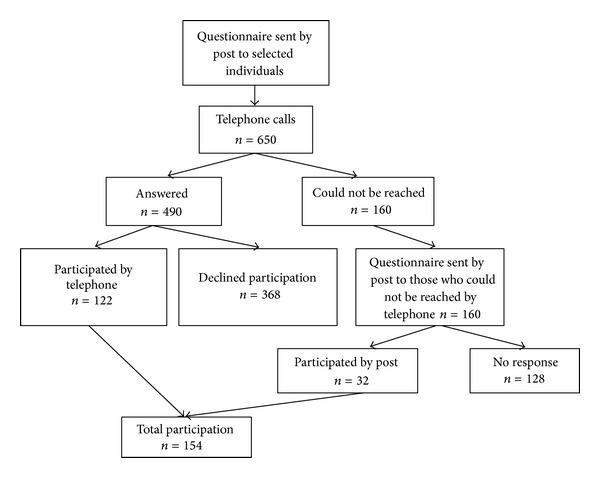
Flowchart of the inclusion process.

A total of 650 individuals aged 55–105 were randomly selected from the official identity and address registry for Swedish residents [20]. As shown in the flowchart of the inclusion process (Figure 1), a total of 154 persons responded to the survey, 368 declined, and 128 could not be reached. The age range for those who participated in the study was 55 to 91 years. A sample failure analysis showed that respondents differed from nonrespondents regarding age (mean respondent age was 71.9, while mean nonrespondent age was 74.1) (*P* = 0.010) but not with regard to gender (*P* = 0.407). The profile of the respondents is presented in Table 1.

### 2.2. Instrument

**Figure 2:**
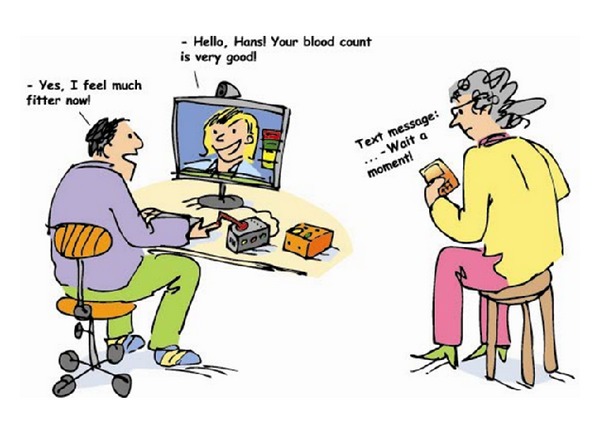
Web-based e-health services in the present.

**Figure 3:**
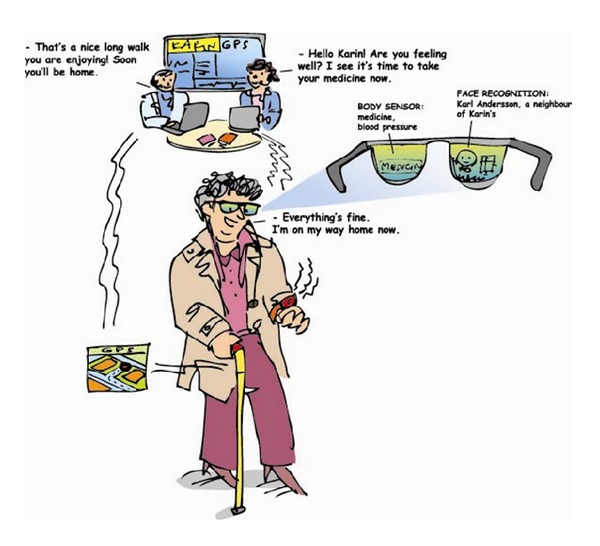
Mobile health applications in the future.

The two illustrated scenarios of this study's survey were based on focus group discussions from the research project MyHealth@Age (2008–2010) [21, 22] developed with a researcher focusing on pervasive and mobile computing at the Luleå University of Technology and ultimately, thereafter designed by a graphic designer. A scenario can be defined as a description of a possible set of events that might reasonably take place; for our survey, scenarios focus on use, what people can do with a system, and the consequences for users [23]. The present scenario illustrates existing web-based e-health services together with an explanatory text of how people are able to use web-based e-health services, if needed (see Figure 2). The future scenario illustrates mobile health applications under development together with an explanatory text of how people in the future will be able to use mobile health applications, if needed (see Figure 3).

#### 2.2.1. Present Scenario

Presently and within the next few years, you can/are able to, if necessary,

- renew your prescriptions and book appointments at health care centers on the Internet,
- receive SMS appointment reminders,
- receive advice from the online medical counseling service,
- contact health care staff by email,
- take your blood pressure, ECGs, and blood tests at home by yourself and send the results via the Internet to health care centers,
- talk to health care staff about your test results using a web camera,
- receive advice on how you should exercise and what you should eat to maintain or attain good health.

#### 2.2.2. Future Scenario

In the future, you will be able to, if necessary,

- be in constant contact with a health professional via sensors that will alert your health care center if they detect any problems with a measured value,
- track your fitness improvement by measuring walking distance, pulse, and blood pressure automatically,
- recognize people and receive assistance in remembering their names with the help of special glasses,
- wear a personal safety alarm that can determine your exact position in the event of you fall outdoors and need assistance,
- use a walking stick that shows you the way,
- use sensors in your shoes to obtain better balance.

In responding to the illustrated scenarios (Figures 2 and 3) the Psychosocial Impact of Assistive Devices Scale (PIADS) questionnaire [24] was used to measure the anticipated impact on psychosocial factors of present web-based e-health services and future mobile health applications. The PIADS measures aspects related to quality of life that refer both to the person and the environment [14]. The scale is designed to assess the experienced or anticipated impact of assistive technological devices before using them, thus anticipating the successful use or rejection assistive technologies [25]. PIADS has good internal consistency as well as strong construct and predictive validity [25–27]. The questionnaire can be administered individually, in a group, or via a telephone interview [24]. It consists of 26 items based on the user's description of how devices impact quality of life. Each item is rated on a scale ranging from −3 (i.e., maximum negative impact) to +3 (i.e., maximum positive impact). Ratings are presented as three separate subscores that describe user perceptions along three dimensions (i.e., competence, adaptability, and self-esteem), as well as a total score. The competence dimension is evaluated by questions concerning topics such as competence, productivity, usefulness, performance, and independence. The adaptability dimension is evaluated by questions concerning topics such as the ability to participate, willingness to make changes, eagerness to try new things, and ability to take advantage of opportunities. The self-esteem dimension is evaluated by questions concerning topics such as self-esteem, security, sense of power and control, and self-confidence [14].

The survey's background questions asked for respondents' age, gender, marital or cohabitation status, education level, income, self-rated health status according to a visual analog scale ranging from 0 to 100 and derived from the EQ-5D [28], and experience using ICT applications during a one-year period. Other questions inquired about use of the Internet for obtaining information about health, physical activity, and diet, while other questions asked respondents to report their experience using ICT for health care services (see Table 1). A pretest, with three older adults who responded to the survey and provided information including an information letter, background questions, and scenarios with PIADS, was conducted. The pretest clarified the importance of focusing on anticipated events as opposed to experiences with web-based e-health services at the present and mobile health applications in the future when rating PIADS. A logbook was used by the telephone interviewers (i.e., the first author and an assistant) to collect spontaneous comments and feedback from respondents.

### 2.3. Procedure

The survey was distributed by post to 650 randomly selected individuals in batches of 100. After the expected delivery date, recipients were contacted by telephone. This process was repeated until all 650 surveys were distributed. Upon a recipient's consent to participate, a telephone interview was immediately conducted. First, a set of background questions were answered (see Table 1). Respondents were then asked to study the illustration of the web-based e-health services in the present scenario and read the written explanation in order to rate their anticipated psychosocial impact concerning the 26 items in the PIADS. The same procedure was repeated for the future scenario illustrating mobile health applications. During the telephone interviews, the comments of respondents were noted by the interviewers in a logbook. Recipients who declined to participate were asked to provide data regarding their gender and age. Recipients who could not be reached after five attempts by telephone were mailed a survey including a glossary to help them interpret the PIADS items (cf. [27]) and asked to complete and return the survey to researchers using prepaid, preaddressed envelopes.

### 2.4. Analyses

Parametric analyses were made for the variables age and self-rated health because they were ratio variables and normally distributed. All other variables were analyzed nonparametrically, as they were at either nominal or ordinal levels, or, as in the case of the PIADS scores, presented a skewed distribution. For analyses of the statistical inference of differences between two groups, a Student *t*-test was used for the parametric dependent variables, a Mann-Whitney *U*-test for ordinal variables, and the *χ* ^2^-test for nominal variables (see the description of sampling failure analysis on page 5 and Tables 3 and 4). When analyzing the differences in PIADS scores between the present and future scenarios (see Table 2), the Wilcoxon signed-rank test was used because of its dependent measurements. Correlations between PIADS total scores and parametric and ordinal scale variables were analyzed by the nonparametric Spearman's rank correlation (see Tables 3 and 4). All analyses followed standard procedures and considerations [29], and the significance level was set at 5%.

## 3. Results

As shown in Table 2, data indicated that respondents anticipated positive psychosocial impacts for using web-based e-health services and mobile health applications regarding both present and future scenarios. Even first quartile values were consistently positive. Only 19 respondents (12%) reported negative total PIADS scores for the present scenario and 14 (9%) for the future scenario (data not shown). More importantly, both total PIADS score and sub-scores were significantly higher for the future scenario than those for the present. Among all scores, the adaptability sub-score showed the highest values.

Age was significantly related to the total PIADS scores regarding both web-based e-health service and mobile health application scenarios (see Tables 3 and 4), while gender, marital or cohabitation status, income, and educational levels were not. General ICT experience (mobile phone, SMS, computer use, email, and Internet use) was consistently and significantly associated with the psychosocial impact anticipated from the present scenario, but less consistently for the future scenario. At the same time, self-related health was associated with the anticipated psychosocial impact for the future scenario but not the present. Experience with health related ICT (SMS, email, web, chat, blog, and audiovideo communication) was not shown to be associated with the anticipated psychosocial outcome of either scenario except regarding using the Internet to retrieve health information in the present scenario.

## 4. Discussion

For both scenarios, a pattern of positive anticipation for the psychosocial impact of using web-based e-health services and mobile health applications is clear. Mobile health applications resulted in a higher anticipated psychosocial impact when compared to web-based e-health services. Contrary to our expectations, respondents did not rate more highly the anticipated psychosocial impact of the present scenario despite being familiar with its description of the services currently available in Sweden [6, 30, 31]. On the whole, it seems that older adults may find meaning from the perspective of the usefulness [17] of mobile health applications. The future scenario could be interpreted to suggest situations of independence and sociability for the mobile health application illustrate the transition of e-health services towards mobility and self-management.

Adaptability received the highest PIADS sub-score for both scenarios, particularly for mobile health applications in future scenario. Such a result suggests that these tools are more related to the environment in the sense that mobile health applications can serve as adapters that liberate users and enable them to pursue activities in daily life [14]. Independence was found to be imperative among older adults in order to avoid social exclusion [32] and is, in the sense of control and choice, of great importance when older adults use e-health services [33]. In one study [13], older adults with functional limitations reported that they were afraid of losing control and that control was strongly connected to feelings of independence. From the logbook, we read the following comments about scenario representing mobile health applications. “It would be fantastic if I could get that kind of help if I needed it;” “Do you really mean that this is being developed?,” and “Yes, it would be helpful.” This scenario painted respondents a picture of the technology being adapted to users instead of users being adapted to the technology and was perceived to be well integrated and will allow users autonomy without feeling intruded upon (cf. [9, 34–37]). These results could in turn reduce stigmatization (cf. [9, 38]). Mobile applications thus seem to be an important service that promotes mobility, which in turn positively impacts the quality of life of older adults [39].

The correlation between increased age and lower anticipated psychosocial impact may be expected, as previous research has shown a lower usage of ICT and a decreased interest in ICT communication with health care services at very old ages [31, 40]. The decreased psychosocial anticipations with increased age may be explained by the fact that the oldest adults perceive technical devices to be uninteresting because of low self-efficacy in relation to ICT use [41].

Results also showed a connection between general ICT use and the anticipation of web-based e-health services in the present scenario, as well as a connection between the use of SMS messaging and the Internet with the anticipation of mobile health applications in the future scenario. This result may confirm that web-based e-health services to some extent possess an image recognition factor similar to general usage. The mobile health applications in the future scenario, SMS messaging, and the Internet may be considered by the respondents to be more advanced, thus making the connection somewhat obvious.

Internal validity was strengthened by survey pretests, low internal data losses, a glossary explaining the PIADS items, and a standardized procedure during interviews. A few days after the first delivery of 100 surveys, all authors contacted five individuals by telephone for a total of 20 older adults in order to investigate the possibility that recipients needed clarification on the survey and interview procedures.

In the area of e-health, there are, to our knowledge, no instruments for evaluating psychosocial outcomes. We had to search in comparable areas and for assistive technology, there are a plethora of scales for assessing the impact and outcomes of devices. PIADS was developed and used in the context of assistive technology as wheelchairs, hearing aids, and so forth [42–44]. Quebec User Evaluation of Satisfaction with Assistive Technology (QUEST 2.0) assesses users' satisfaction of a device and assumes that an experience takes place with the device and that was not the case in our study [45]. Another one is Matching Person & Technology (MPT), an instrument that helps professionals together with the consumer, based on the person's goals, identify technologies which are desired and needed but not yet available [46]. MPT assesses the problems and barriers for use rather than anticipated psychosocial impact. We chose to use PIADS as an instrument because the design of this instrument fits our purpose, to evaluate the anticipated psychosocial impact of the e-health scenarios described. E-health means tools and services using information and communication technologies to assist the user, with it being important to measure the anticipated impact that these tools and services will have on the lives of older users and their environment. This study marks the first time that this instrument has been used to investigate the anticipated psychosocial impact of e-health services. We consider PIADS to be a valid instrument for this purpose, as it has been used to evaluate other technologies that support personal health [42–44].

We attempted to reduce the rate of nonresponses by making several telephone calls to recipients using a two-mode strategy (i.e., postal delivery and telephone interviews). Doing so may have positively affected the response rates. At the same time, it is possible that the number of questions negatively affected response rates. Other negative factors may include a lower interest level in the survey issues, health issues, and surveys in general [47]. Some recipients who declined to participate provided the following comments during telephone conversations: “I am too old to be answering this;” “I am not interested;” and “I am too sick.” Other nonrespondents found the questions to be excessive in number or too difficult to answer.

Prestudy sample size calculations showed that in order to detect differences in PIADS scores between two unequally sized groups corresponding to at least moderate effect size, considering a significance level of 0.05 and a statistical power of 0.80, at least 319 respondents would be necessary. Considering an expected nonresponse rate of 50%, we therefore asked 650 persons to participate. Despite serious efforts to the contrary, the nonresponse rate was ultimately higher than expected (74%), which is an external validity threat for implying a potential nonresponse bias. This result also highlights possible type-II errors regarding *P* values bordering on statistical significance (see, e.g., Table 4). We nevertheless believe that our findings are of interest. First, the sample was derived from random selections of the official Swedish population registry. Second, we have reason to believe that the nonresponse bias may not have been too critical. Our data did not indicate large selection bias concerning gender and age, and the general ICT experience in our sample did not differ substantially from previously published results [31]. Although high response rates are preferred to ensure sample-to-population representativeness, empirical data seem to show that low response rates are not necessarily connected to large bias [48]. Third, as the PIADS scores were consistently positive to a high degree, we could expect that a possible overestimation resulting from sampling bias is less likely to distort the overall picture.

## 5. Conclusion

We found that the anticipated psychosocial impact was positive for web-based e-health applications in the present scenario and also for mobile health applications in the future scenario but was negatively correlated to an increase in age. Such findings may be interpreted to be especially interesting and unique since they concern an entire population of older adults and are not limited to specific diagnostic groups or samples participating in ICT trials. By contrast, our findings indicate that in a population of older people, e-health is likely to positively impact quality of life from a psychosocial perspective. Considering these aspects can serve as an important contribution to facilitating technology diffusion in health care among older adults. In the future, we can expect a continued increase in general ICT experiences among older adults, including the oldest, which will perhaps decrease the effects of advanced age on the anticipated impact. As long as differences persist, however, we must acknowledge the possible digital divide when implementing e-health. For the oldest members of the population, it is important to investigate their needs from a psychosocial perspective in order to tailor health care services that are meaningful in their particular context.

## Figures and Tables

**Table 1:** Profile of respondents (*n* = 154).

	*n* (%)	Mean (SD)	*n* (no/yes)*	Md (q1, q3)***
Age		71.9 (±8.7)		
Female gender	80 (52)			
Living alone	49 (32)			
Education level				
Primary school	55 (36)			
College	50 (34)			
University	47 (31)			
Monthly income (SEK)				
<8,000	14 (9)			
8,000–18,000	71 (47)			
18,000–26,000	42 (28)			
>26,000	25 (16)			
Self-rated health (VAS 0–100)		72.5 (±18.0)		
Usage degree of mobile phone**				3.0 (2.0, 4.0)
Usage degree of SMS messaging**				1.0 (1.0, 2.0)
Usage degree of computer**				3.0 (1.0, 4.0)
Usage degree of e-mail**				2.0 (1.0, 3.0)
Usage degree of Internet**				3.0 (1.0, 4.0)
Used Internet for health information*			95/58	
Used Internet for information about physical activity*			130/23	
Used Internet for information about diet*			117/36	
Used mobile phone or advanced ICT in contacts with health care*			103/50	

*Self-reported usage of mobile phone, SMS, e-mail web, chat, blog, and audiovideo communication during a one-year period. **Self-rated ordinal scale for general ICT experience during a one year period: 1 = never; 2 = monthly; 3 = weekly; 4 = daily ***Md: median; q1: lowest quartile; q3: highest quartile.

**Table 2:** PIADS total score and subscores for web-based e-health services in present and mobile health applications in the future.

Variable	Present* Md (q1, q3)***	Future** Md (q1, q3)***	*P* value
Total score (*n* = 147/150)	0.81 (0.27, 1.23)	1.00 (0.46, 1.54)	<0.001
Competence sub-score (*n* = 149/150)	0.75 (0.29, 1.34)	1.00 (0.42, 1.52)	0.002
Adaptability sub-score (*n* = 150/150)	0.84 (0.34, 1.50)	1.17 (0.50, 1.83)	<0.001
Self-esteem sub-score (*n* = 149/150)	0.75 (0.13, 1.13)	0.88 (0.38, 1.50)	0.001

*Present = describing a scenario where web-based e-health services are able to be used in present. **Future = describing a scenario where mobile health applications will be able to be used in the future ***Md: median; q1: lowest quartiles; q3: the highest quartiles.

**Table 3:** Associations between PIADS total score for web-based e-health services in present scenario and respondents' profile variables (*n* = 147).

Independent variable	Mean rank difference	*r*_*s*_	*P* value
Age		−0.217	**0.008**
Gender (female/male)	−4.5		0.520
Living alone/together with someone	−7.9		0.292
Education level		0.056	0.502
Income		0.113	0.174
Self-rated health (VAS 0–100)		0.110	0.184
Usage degree of a mobile phone**		0.167	**0.043**
Usage degree of SMS messaging**		0.270	**0.001**
Usage degree of a computer**		0.180	**0.029**
Usage degree of emails**		0.202	**0.014**
Usage degree of the Internet**		0.227	**0.006**
Used the Internet for health information (no/yes)*	−16.61		**0.021**
Used the Internet for information about physical activity (no/yes)*	−16.03		0.097
Used the Internet for information about diet (no/yes)*	−10.84		0.189
Used mobile phone or advanced ICT in contacts with health care*	−6.79		0.360

*Self-reported usage during a one-year period (no/yes). **Self-rated ordinal scale for ICT experience in a one-year period: 1 = never; 2 = monthly; 3 = weekly; 4 = daily; *r*_*s*_: Spearman's rank correlation coefficient.

*P* value: statistical significance.

Bold: values below significance level 0.05.

**Table 4:** Associations between PIADS total score for the future mobile health applications scenario and respondents' profile variables (*n* = 150).

Independent variable	Mean rank difference	*r*_*s*_	*P* value
Age		−0.212	**0.009**
Gender (female/male)	2.1		0.763
Living alone/together with someone	−4.0		0.600
Education level		0.002	0.984
Income		0.107	0.192
Self-rated health (VAS 0–100)		0.186	**0.022**
Usage degree of a mobile phone**		0.135	0.100
Usage degree of SMS messaging**		0.230	**0.005**
Usage degree of a computer**		0.124	0.132
Usage degree of emails**		0.202	0.165
Use degree of the Internet**		0.162	**0.048**
Used the Internet for health information (no/yes)*	−11.72.		0.107
Used the Internet for information about physical activity (no/yes)*	−19.16		0.052
Used the Internet for information about diet (no/yes)*	−15.51		0.064
Used mobile phone or advanced ICT in contacts with health care	−2.62		0.727

*Self-reported usage during a one-year period (no/yes). **Self-rated ordinal scale for ICT experience in a one year period: 1 = never; 2 = monthly; 3 = weekly; 4 = daily.

*r*_*s*_: Spearman's rank correlation coefficient.

*P* value: statistical significance.

Bold: values below significance level 0.05.
